# Two siblings with immunodeficiency, facial abnormalities and chromosomal instability without mutation in DNMT3B gene but liability towards malignancy; a new chromatin disorder delineation?

**DOI:** 10.1186/1755-8166-3-5

**Published:** 2010-03-08

**Authors:** Anna Polityko, Olga Khurs, Natalia Rumyantseva, Irina Naumchik, Nadezda Kosyakova, Holger Tönnies, Karl Sperling, Heidemarie Neitzel, Anja Weise, Thomas Liehr

**Affiliations:** 1 National Medical Center "Mother and Child", Orlovska Street 66, 220053 Minsk, Republic of Belarus; 2 Jena University Hospital, Institute of Human Genetics and Anthropology, Kollegiengasse 10, D-07743 Jena, Germany; 3 Institute of Human Genetics, Charité, Humboldt-University, Augustenburger Platz 1, 13353 Berlin, Germany; 4 Robert Koch Institute, Nordufer 20, 13353 Berlin, Germany

## Abstract

**Background:**

ICF syndrome (standing for Immunodeficiency, Centromere instability and Facial anomalies syndrome) is a very rare autosomal recessive immune disorder caused by mutations of the gene de novo DNA-methyltransferase 3B (DNMT3B). However, in the literature similar clinical cases without such mutations are reported, as well.

**Results:**

We report on a family in which the unrelated spouses had two female siblings sharing similar phenotypic features resembling ICF-syndrome, i.e. congenital abnormalities, immunodeficiency, developmental delay and high level of chromosomal instability, including high frequency of centromeric/pericentromeric rearrangements and breaks, chromosomal fragments despiralization or pulverization. However, mutations in DNMT3B could not be detected.

**Conclusion:**

The discovery of a new so-called 'chromatin disorder' is suggested. Clinical, molecular genetic and cytogenetic characteristics are reported and compared to other 'chromatin disorders'.

## Background

Several human diseases which are characterized by alterations or modification of chromatin structure caused by changes of methylation pattern are considered as so-called 'chromatin disorders' [1]. Their delineation and the encoding of mechanisms underlying these genetic syndromes is one of the ways to understanding the global principles of functioning of genomic DNA and chromatin in human. ICF syndrome (for immunodeficiency, centromere instability and facial anomalies; OMIM #242860) belongs to the aforementioned disorders and is a rare recessive disease caused by mutations of the gene DNMT3B that encodes the 'de novo DNA-methyltransferase 3B' [2-4]. Patients with ICF syndrome demonstrate immunodeficiency, facial anomalies, mental retardation and developmental delay. The main clinical feature is reduced serum immunoglobulin levels that lead to death due to severe recurrent infections, often before adulthood. The typical cytogenetic markers of ICF syndrome are distinctive 'undercondensation' of heterochromatic segments of chromosomes 1, 9, and 16 and multibranched configurations of those. The chromosomal instability observable in PHA-stimulated lymphocytes correlates with a severe hypomethylation of the classical satellites 2 and other genomic sequences such as alpha satellites, the centromeric component of constitutive heterochromatin 3, Alu sequences D4Z4 and NBL2 repeats and certain imprinted genes [5].

Recent data showed that clinically defined ICF patients divided into two subgroups and only about 60% of them had the DNMT3B mutations and normal methylation of the alpha satellites. Those ICF patients without the typical mutation showed hypomethylation of the alpha satellites [5-7]. For both groups is in common, that they have characteristic heterochromatin abnormalities and undermethylation of classical satellites 2 and 3.

Here we report on a family with ICF-like symptoms but without DNMT3B mutation and with a rather untypical pattern of heterochromatin abnormalities.

## Results

### Case report

Mother and father were not related and physically and intellectually healthy. The first gestation of this partnership ended with a spontaneous abortion (no further information available). After uncomplicated second and third full-term pregnancies two girls were born, designated as P1 and P2 in the following. Additionally, there is a half-sister of P1 and P2, a child of father's first marriage, who was healthy.

P1 was born when father and mother were 33 and 34 years old, respectively, with a birth weight of 2200 g (<3rd centile), length of 45 cm (<3rd centile), and an OFC of 31.5 cm (<3rd centile). P2 was born 4 years later and had a birth weight of 1900 g (<3rd centile), a length of 43 cm (<3rd centile), and an OFC of 31 cm (<3rd centile). P1 and P2 showed severe pre- and postnatal growth delay which persisted in the following. At the age of 6 years P1 had a weight of 13 kg (<3rd centile), a height of 110 cm (= 10 centile) and an OFC of 46.5 cm (<3rd centile). Similarly P2 had at 1 year a weight of 6 kg (<3rd centile), a height of 62 cm (<3rd centile) and an OFC of 42 cm (<3rd centile). Both sisters suffered from recurrent respiratory infections, immunodeficiency, anemia and mild mental retardation. They had concordant phenotypic features: microcephaly, craniofacial features included "bird-like" appearance, hypertelorism, low-set ears, epicanthal folds, arched palate, microretrognathia, eye abnormalities (P1 - strabismus, myopia; P2 - hypermetropic astigmatism), chest deformations, arachnodactyly and plano-valgus feet. Renal and brain defects were not found by ultrasound examination. Biochemical screening for metabolic diseases were normal in both of cases. Finally, P1 had cardiomyopathia starting at the age of 2 years and a thrombocytopenia at age of 4 years. With the age of 6 years she developed an unspecified type leukemia, from which she died at age of 8 years and 2 months; the bone marrow cells karyotype was complex and included abnormal clones harboring monosomy 7 and other constitutional aberrations like deletion in chromosomes 1, 5, 6 and 13: The leukemia karyotype was as follows: mos 45, XX,-7/45, idem, del(13)(q11q22)/46, XX, del(1)(p35)/46, XX, del(5)(q12q33), del(6)(q13q25). Cytogenetics of peripheral blood of P1 and P2 as well of the parents was done at this time and the 'chromatin disorder' detected (see below).

Furthermore, the DNMT3B DNA methyltransferase gene mutated in the ICF immunodeficiency syndrome [5-7] was sequenced in P1 and P2 and no hint on a mutation was detected, thus, excluding a common ICF syndrome. In detail the following polymorphisms were detected leading to no effect on the aminoacid sequence: in exon 1: NT_028392.4 position 1534716; in exon 15: NT_028392.4 position 1553115 and NT_028392.4 position 1553217; in intron IVS18-75: NT_028392.4 position 1555777, in intron IVS17-5: NT_028392.4 position 1555404, in intron IVS16-5. NT_028392.4 position 1554722; in intron IVS09-113: NT_028392.4 position 1547997; in untranslated RNA region 5'mrnautr: NT_028392.4 position 1562920. Moreover, in exon 9 at ENST00000328111 position 30355 the sequence was altered in a way that a leucin instead of an isoleucin is inserted in the DNMT3B gene product. However, also this alteration is classified as a harmless polymorphism in 'Human Mutation Database' http://www.hgmd.cf.ac.uk and 'Mutation Taster' http://neurocore.charite.de/MutationTaster/comparison.html.

Unfortunately, the family was lost during further follow-up. Nonetheless, Epstein Barr virus based immortalization of the parental blood and blood of P1 and P2 was done [8]. Thus, cell lines are available for further research.

### Cytogenetic and molecular cytogenetic studies

Karyotypes (GTG-banding) of the siblings P1 and P2 and their parents were normal. Chromosomal stability of P1 and P2 and their parents were tested by analysis of GTG-banding or standard solid-stained chromosomes using 48, 72 and 96 h blood lymphocyte cultures as presented in Table 1.

**Table 1:** The chromosome instability of peripheral blood lymphocytes in P1, P2, and their parents, after studying by conventional and banding cytogenetic methods

Patient	Age at the time of blood sampling	Culture/ chromosome staining	Totally cells scored	Aberrations frequency (%)
P 1	5 years 6 months	72 h/solid (standard)	147	23 (15.6 ± 3.16)
	
	6 years 2 months	48 h/solid (standard)	132	32 (24.24 ± 3.73)
	
	6 years 2 months	48 h/GTG	253	83 (32.81 ± 2.95)
	
	7 years 10 months	72 h/GTG	306	68 (22.22 ± 2.38)

P 2	1 year 8 months	96 h/GTG	35	14 (40.0 ± 8.28)
	
	4 years 4 months	72 h/GTG	200	19 (9.5 ± 2.77)

Mother	39 years	72 h/GTG	197	6 (3.05 ± 1.31)

Father	38 years	72 h/solid standard	200	16 (8.0 ± 1.92)
	
	41 years	72 h/GTG	100	4 (4.0 ± 1.96)

The chromosomal stability in parents of P1 and P2 was considered as normal, besides a previously unrecognized hint of a specific low level of mosaic aneuploidy of X-chromosome in the father (5 of 100 cells with 47, XXY). This was studied by two-color interphase FISH using centromeric probes for the X- and the Y-chromosomes (Abbott/Vysis) in 670 cells: 4 cells had abnormal sex chromosome constitution (1 times 45, X; 3 times 47, XXY). Thus, the presence of a minimal mosaic 47, XXY/46, XY in the father could not be excluded.

**Figure 1:**
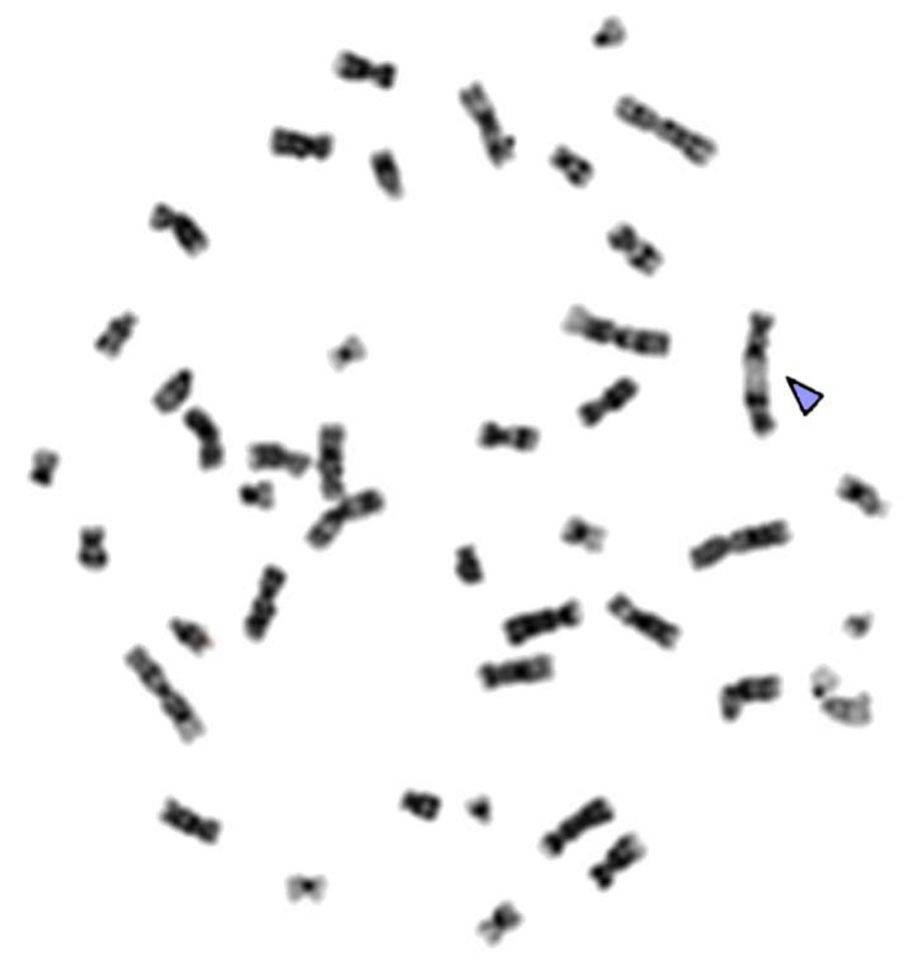
**Example of a metaphase analyzed by conventional method of aberration scoring: An end-to-end junction of chromosomes 12 is marked by arrowhead**.

**Figure 2:**
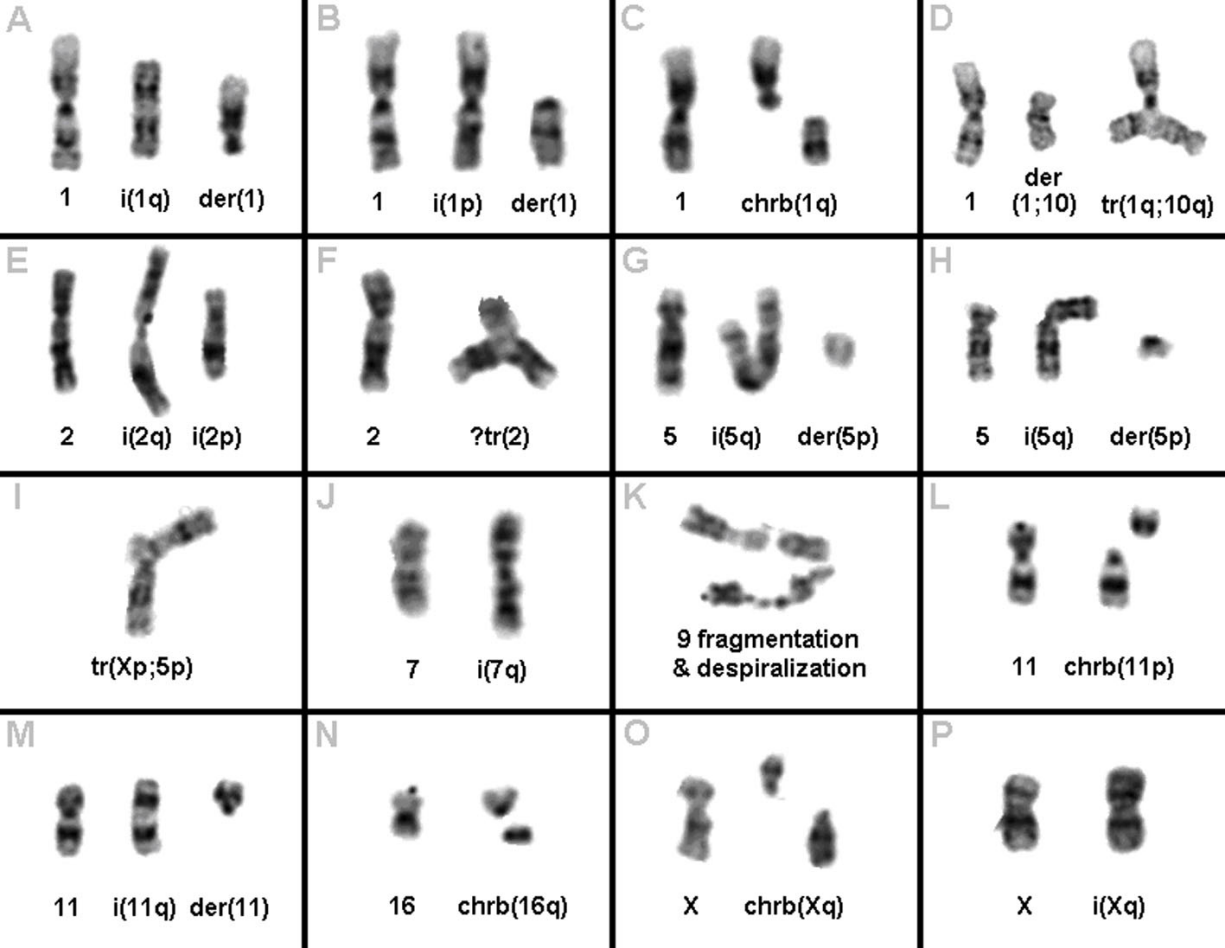
**Chromosome aberrations detected by GTG-banding in P1 and P2**: A-C) Aberrations including chromosome 1 like isochromosomes, derivative chromosomes and chromosome breaks. D) Chromatide exchange between chromosomes 1 and 10 with formation of triradial tr(1;10)(q12;q25). E-F) Aberrations of chromosome 2 like triradial chromosome 2 with 2q-arm duplication and isochromosomes 2. G-H) Isochromosomes 5q together with derivative chromosomes 5p. I) A chromatide exchange between chromosomes X and 5 led to formation of a triradial tr(X;5)(p13;p11~21). J) An isochromosome 7q was formed. K) Despiralization and fragmentation of chromosome 9. L-M) Isochromosome and chromatid-break involving chromosome 11. N-O) Chromatid-break events in chromosome 16 and X. P) Isochromosome Xq-formation.

**Figure 3:**
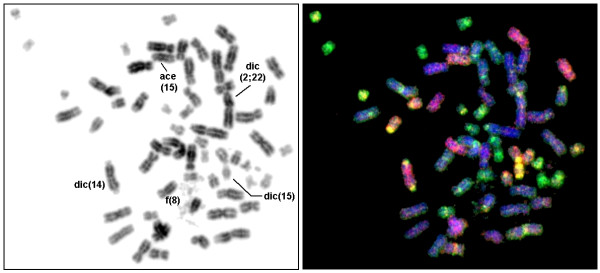
**Chromosome aberrations studied by Multiplex-FISH (M-FISH) method applying whole chromosome painting probes**. Result is depicted in inverted DAPI-banding on the left and after M-FISH on the right. The near-triploid (hypotriploid) metaphase carried 60 chromosomes and multiple aberrations including fragmented chromatin belonging to chromosome 8 (= f(8)), dicentric chromosomes like a dic(14;14), a dic(15;15), and a dic(2;22), accompanied by acentric fragments (e.g. ace(15)).

The cytogenetic findings in somatic cells of both the affected girls (P1 and P2) were similar; i.e. high level of unspecific spontaneous chromosomal aberrations were present: chromatide and chromosome type deletions, dicentric and ring chromosomes, reciprocal and nonreciprocal translocations, (small) marker chromosomes, end-to-end chromosomal junctions, aneuploid, poliploid cells and other were found (Figs. 1 and 2). Specific for our patients were especially centromeric rearrangements: high frequency of breaks in centromeric region, isochromosomes, frequently metaphases contained isochromosome and the rest of another arm, additional whole-size chromosomal arms in metaphases, interchanges between chromosomes in pericentromeric regions, fragmentation and/or despiralization of the chromatin. Multiplex-fluorescence in situ hybridization (M-FISH) analysis [9] was applied for further investigation of chromosomal specificity of cytogenetic instability in these family members (Table 2). Some aberrations as detectable by M-FISH analysis are presented in Fig. 3.

**Table 2:** The chromosomal aberrations per 100 peripheral blood lymphocytes of 48 h culture in P1 and P2, and their parents using the M-FISH approach.

Chromosomal abnormalities per 100 cells	Patient P1	Patient P2	Mother	Father
Total number of aberrant metaphases	59	36	5	11

Total number of aberrations	90	43	7	13

Specific centromere-region aberrations	24	17	3	4

Spectrum of chromosomal aberrations				

**Isochromosomes**	4	2	-	1

**Whole arm duplication**	4	1	-	-

**Centromeric breaks**	10	11	2	2

**Chromatine despiralizations/fragmentations**	6	3	1	1

Translocations, inversions	5	2	-	1

Dicentric/ring chromosomes	9	3	-	1

Chromosome deletions, acentrics	28	11	1	5

Chromatide deletions	8	6	2	1

Chromosome exchanges	5	-	-	-

Telomeric junctions	1	1	-	-

Aneuploid/polyploid cells with marker chromosomes	10	3	1	1*

*47, XXY

**Figure 4:**
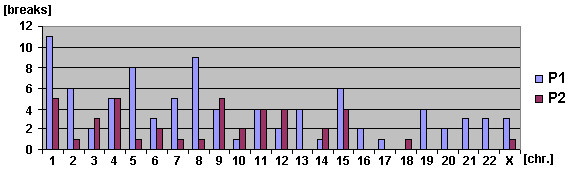
**Distribution of the chromosomes involved in aberrations in P1 and P2**.

Using M-FISH approach we obtained data of chromosomal origin in cases of undercondensation, fragmentation or pulverization of abnormal chromatin. M-FISH was helpful in analysis of near-triploid and near-tetraploid metaphases bearing multiple constitutional aberrations. That made it possible to analyze the involvement of all chromosomes into the aberrations in somatic cells in both of affected children (P1 and P2; see Fig. 4).

## Discussion

The clinical, cytogenetic and molecular genetic status of a family with two siblings having concordant phenotypic abnormalities, developmental delay, immunodeficiency and highly expressed chromosomal breakage are reported. 72 h and 96 h cell cultures were applied for aberration frequency evaluation in spite of the fact that there is the elimination of unstable chromosomal aberration in it. The first cytogenetic examination of P1 discovered 15.6% of aberrations in 72 h culture. A second test 8 months later showed 24.2% in 48 h culture using the same standard staining. Banding technique using the same 48 h culture showed 32.8% of aberrations in second test at age 6 years 2 months of P1, and 1 year 8 months later the level was 22.2%. Patient P2 was examined twice using 96 h and 72 h cultures and GTG -banding method. She showed 40% of aberrations at age 1 year 8 months, and 2 years 4 months later 9.5%.

The accuracy of M-FISH analysis allowed characterizing precisely the levels of chromosomal breakage; abnormal metaphases and juxtacentromeric aberrations (Tab. 2). Evident specificity of chromosome instability was presented by high frequency of centromeric/pericentromeric rearrangements and breaks, chromosomal fragments despiralization or pulverization.

Mentioned phenotypic and cytogenetic characteristics alluded to the rare ICF syndrome, however, both of siblings had not detectable mutation in the DNMT3B gene but only the identical polymorphisms were found. Remarkably, cytogenetically a pronounced prevalence of chromosomes 1, 9 and 16 into the aberration involvement in both of siblings was not detectable, though, chromosome 1 was the most unstable chromosome in patient P1 (Fig. 4). Also we did neither discover an undercondensation of heterochromatic regions of chromosomes 1, 9, and 16 nor classical multibranched chromosome configuration (chromosomal "stars") also reported as cytogenetic markers of ICF-syndrome. At the same time, characteristic aberration at centromeric/pericentromeric regions (isochromosomes, whole arm duplication, centromeric breaks, chromatine despiralizations/fragmentations) were remarkably frequent in P1 (24.0%) and P2 (24.6%), and even observable at a low rate in healthy mother (3.0%) and father (6.7%) - see Tab. 1. Thus, it can be speculated, that the unknown gene in siblings and both parents resulted in increased aberration levels.

## Materials and methods

### Cytogenetics

For investigation of frequency and spectrum of aberrations metaphase chromosomes were obtained according to standard procedures using PHA stimulated 48, 72 and 96 h cultures of peripheral blood lymphocytes from the P1, P2 and parents of both. The analysis of GTG-banded or standard solid-stained chromosomes was carried out according to standard procedures. Epstein Barr virus based immortalization of the parental blood and blood of P1 and P2 was done as previously reported [8].

### Molecular cytogenetics

To clarify the composition of complex aberrations, irresolvable by conventional banding methods molecular cytogenetic technique was performed: M-FISH using the 24 human whole chromosome painting probes was carried out for the analysis of chromosomal regions and chromosomes most frequently involved in aberrations. Slide preparation, in situ hybridization and cytogenetic analysis were performed as previously described [9]. 100 metaphases were analyzed per individual. Two-color FISH using centromeric probes for X- and Y-chromosomes (Abbott/Vysis) was done according to standard procedures for 2-color-FISH experiments.

## Competing interests

The authors declare that they have no competing interests.

## Authors' contributions

AP, OK, NR, IN and NK performed the cytogenetic studies in the present cases and collected the data relative to this report. AP and TL did the molecular cytogenetic analysis and interpretations. HT, KS and HN were involved in the EBV-immortalization procedures and sequencing. KS and AW did analysis and interpretation of the DNMT3B sequence. AP and TL drafted the paper and all authors contributed to the finalizing of the paper and all authors read and approved the final manuscript.
